# Dystrophin immunogenicity and requirement in myogenic cells: Paradigm shift in gene therapy for DMD

**DOI:** 10.1002/ctm2.1122

**Published:** 2022-11-24

**Authors:** Dariusz C. Górecki

**Affiliations:** ^1^ School of Pharmacy and Biomedical Sciences University of Portsmouth Portsmouth UK

1

Successful genetic treatment for spinal muscular atrophy (SMA) has reawakened hope for a similar breakthrough in another debilitating and lethal neuromuscular disorder, Duchenne muscular dystrophy (DMD). Unfortunately, three recent DMD gene therapy trials were disturbed by severe adverse events (myositis). These drastically different outcomes result from significant molecular differences and should initiate a re‐evaluation of current approaches to the treatment of DMD. Importantly, such adjusting can lead to more effective therapies.

Both SMA and DMD are monogenic disorders leading to progressive muscle dysfunction and deaths. Although there are several types of SMA that differ in age of onset and severity, all are caused by mutations in the same survival motor neuron 1 (*SMN1*) gene, leading to a deficiency of the SMN protein, loss of lower motor neurons, and ultimately muscle atrophy. DMD is an X‐linked recessive disease, where mutations in the *DMD* gene result in the loss of dystrophin and progressive muscle atrophy. Diagnosis is made in early childhood and young adults die due to respiratory and/or cardiac failure.

Treatments for both diseases exploit the re‐expression of missing proteins via exon skipping and gene supplementation. In the first approach, antisense oligonucleotide analogues are used to restore the reading frame and therefore production of functional or semi‐functional proteins (e.g., Nusinersen [Spinraza] for SMA, eteplirsen and golodirsen for DMD), while gene therapy substitutes a healthy copy of the mutant gene (e.g., onasemnogene abeparvovec [Zolgensma] for SMA, GNT0004 for DMD).

Yet, these treatments have been effective in SMA but not DMD. The difference lies chiefly in the absence or presence of the immune response against the re‐expressed protein. Chronic diseases require a continued expression of the therapeutic protein, and it can be immunogenic, which occurs in DMD but not SMA.

The clinical variability in SMA is attributed to the presence of SMN2, a SMN1 paralogous gene, expressing mostly unstable but also small amounts of functional SMN protein (Figure 1). Its presence, albeit insufficient to avert the disease, allows the developing immune system to recognize it as ‘self’. Therefore, the additional therapeutic SMN does not trigger immune responses. Furthermore, the target in SMA is the central nervous sytem (CNS), tissue protected by immune privilege.

**FIGURE 1:**
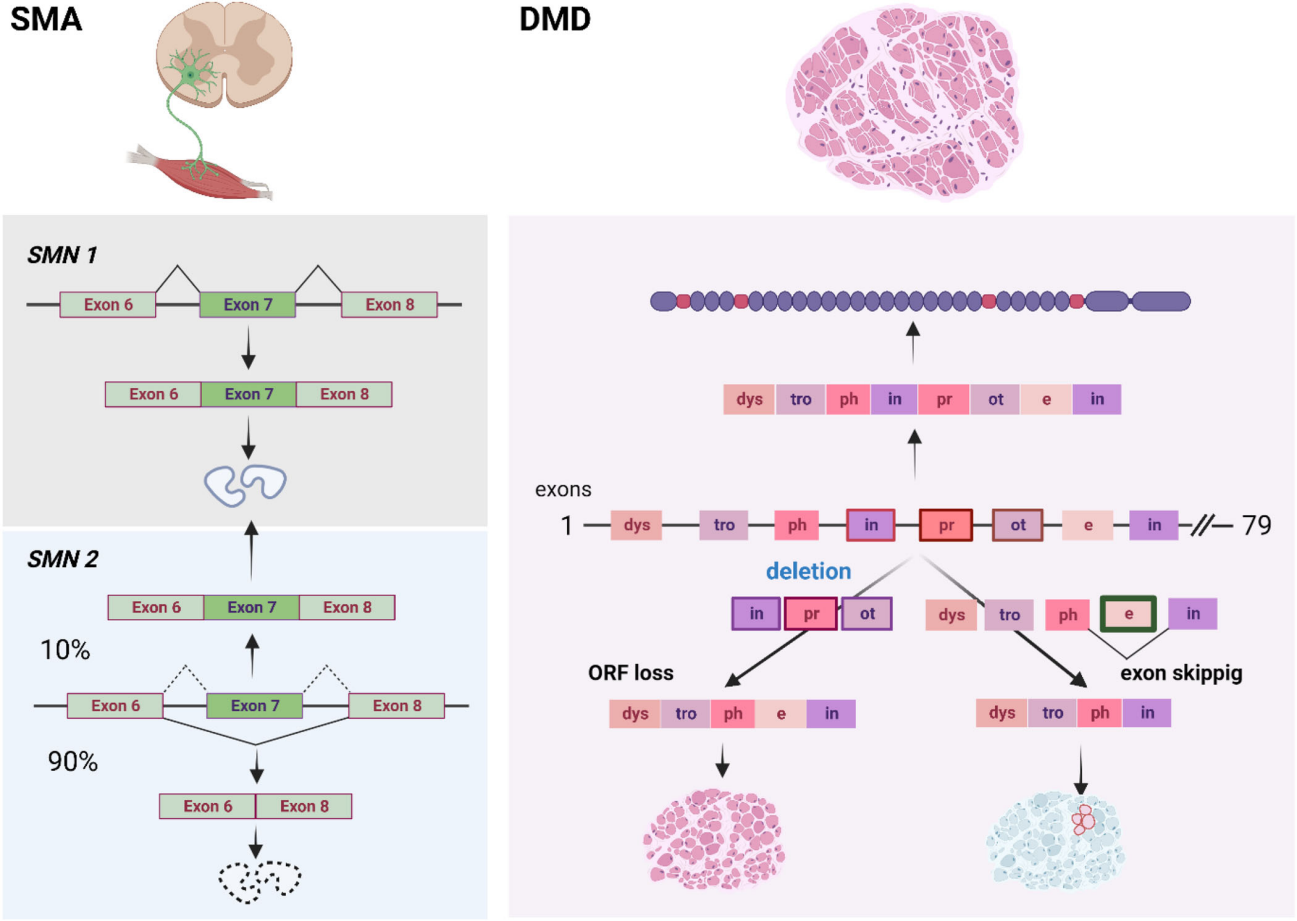
Underlying defects and treatment approaches in spinal muscular atrophy (SMA) and Duchenne Muscular Dystrophy (DMD). In SMA, mutations in SMN1 gene result in SMN protein deficiency, because SMN2 paralogue mostly produces exon 7‐deleted, unstable protein. However, low expression of SMN is sufficient to prevent immunisation when it is re‐expressed therapeutically via the modulation of exon 7 skipping or gene therapy. In DMD, mutations disrupting the open reading frame (ORF) in this largest gene known (79 exons) lead to the absence of dystrophin. Exon skipping is used to restore ORF in a mutation‐dependent fashion and evoke production of internally truncated, semi‐functional minidystrophin. Rare, spontaneous ORF restoration gives rise to revertant fibres. Just like the therapeutically‐expressed one, such minidystrophin is immunogenic and the subsequently re‐expressed therapeutic dystrophin rejection might be accelerated by the memory response.

In contrast, even healthy skeletal muscle is an immunogenic location, as recently exploited in COVID vaccination. Additionally, DMD is associated with chronic muscle inflammation, which augments immunogenicity.

Unfortunately, even small dystrophin epitope differences are recognized as non‐self.^1^ Re‐expressed dystrophin is processed by the antigen‐presenting cells and triggers immune reactions.^1^, ^2^ Dystrophin‐positive muscle become the target for cytotoxic T lymphocytes.^1^ Elimination of transgene‐expressing cells leads to cessation of the therapeutic effect. Worse still, if the level of expression is high and occurs in a large number of myofibers, for example, when efficient targeting vectors are used, the immune response is aggressive and can cause severe damage (myositis), as was the case across recent gene therapy trials. Alas, while the high level of expression is needed to fully protect muscle fibres, the high antigenic load will exacerbate the response.

This unfavourable environment may be further complicated by the appearance of the so‐called revertant fibres in DMD muscles (Figure 1).

Clearly, prevention or suppression of immune responses against re‐expressed dystrophin must be considered. Immunosuppression is likely to be exploited as a short‐term solution. Yet, current immunosuppressive regimens are not free of unwanted effects, including disrupted reactions against pathogens (pulmonary infections, virus reactivation), posing an additional risk to already respiratory handicapped DMD patients. Given the tumour‐suppressor properties of dystrophin,^3^ the malignancy risk in immunosuppressed DMD sufferers must also be considered. Therefore, induction of tolerance to the therapeutic dystrophin would maximize the treatment impact whilst minimizing risks, and such an approach appears vialble.^4^

In addition to immunogenicity, trials aimed at dystrophin re‐expression should consider new pathology findings. Although DMD was known to be active prior to diagnosis, dystrophic defects were recently found early in the development. Moreover, *DMD* mutations affect not only muscle stem (satellite) cells and myofibers but also myoblasts, disturbing key muscle repair functions.^5^, ^6^ Altogether, these findings close the vicious cycle of DMD pathology,^6^ where *DMD* mutations cause stem cell impairment, myoblasts defects reducing muscle regeneration and also formation of unstable myofibers. Surprisingly, while lack of dystrophin during differentiation negatively affects myofiber functional development, dystrophin ablation in fully differentiated myofibers does not trigger their degeneration.^7^, ^8^ Thus, dystrophin re‐expression should be targeted at the myogenic cells rather than myofibers, as it is done currently.

Crucially, re‐evaluation of the current DMD tenets in the light of these new findings offers hope for effective treatments. In contrast to the current therapeutic approaches, targeting dystrophin to myogenic, proliferating cells in younger patients could evoke lasting expression. Not only gene targeting to dividing cells is more effective than to post‐mitotic myofibers, but use of technologies enabling DMD gene repair in muscle stem cells could produce generations of healthy satellite cells and repopulation of muscles with functional myofibers. Simple screening for high serum creatine kinase levels in all new‐born boys would allow perinatal diagnosis of DMD, and application of gene therapy in infants, capable of developing neonatal tolerance.^9^ Early dystrophin re‐expression could prevent dystrophic damage and, even if not curative, it might prevent immune responses against the subsequent treatments. Of course, neonatal tolerogenic protocols could only be considered with a much better understanding of the underlying mechanisms.

Likewise, use of CRISPR/Cas9 technology to repair DMD gene in satellite cells of older patients should be considered with an utmost caution, with effective immunosuppression or tolerance regimens, because it could lead to the immune‐response evoked elimination of muscle stem cells.

Still, exon skipping is only suitable for a small proportion of patients affected by specific mutations, whilst gene therapy, albeit applicable to all, is currently aimed at converting DMD to the milder Becker Muscular Dystrophy (BMD). Furthermore, none of these treatments would target the cognitive and behavioural impairment present in DMD. Given that the development of functional myofibers in the absence of dystrophin appears possible,^10^ an alternative approach is to tackle abnormalities caused by the loss of dystrophin instead of re‐expressing dystrophin. The general principle is identical to the management of inherited diseases such as phenylketonuria with diet or Wilson's disease with copper chelation, where the underlying genetic defect remains, but patients are largely symptoms‐free.

In fact, glucocorticoids are already a gold standard treatment for DMD. Therapeutics reducing inflammation and fibrosis with fewer side effects gave good results in animal models and some entered clinical trials. Promising data were obtained with drugs improving muscle growth and metabolism. Moreover, up‐regulation of the dystrophin homologue utrophin via genetic or pharmacological means could potentially fulfil the same role but without the risk of immune reactions.

The novel approaches to long‐lasting dystrophin restoration combined with those bypassing the loss of dystrophin should result in effective treatments for this devastating disease.

## CONFLICT OF INTEREST

The authors declare no conflict of interest.
